# Study on the Differences in Fecal Metabolites and Microbial Diversity of Jiangshan Black-Bone Chickens with Different Earlobe Colors

**DOI:** 10.3390/ani14213060

**Published:** 2024-10-23

**Authors:** Zhijun Wang, Shiru Li, Xiangying Ding, Xue Du, Ayong Zhao

**Affiliations:** 1 Key Laboratory of Applied Technology on Green-Eco-Healthy Animal Husbandry of Zhejiang Province, Zhejiang Provincial Engineering Laboratory for Animal Health Inspection & Internet Technology, College of Animal Science and Technology & College of Veterinary Medicine of Zhejiang Agriculture and Forestry University, Hangzhou 311300, China; zhijunwang@zafu.edu.cn (Z.W.); lishiru@stu.zafu.edu.cn (S.L.); duxue@zafu.edu.cn (X.D.); 2 Jiangshan Agriculture and Rural Bureau, Quzhou 324100, China; zhaofengershaonv@163.com

**Keywords:** Jiangshan black-bone chicken, earlobe color, metabolome, microbial diversity, melanin

## Abstract

The differences in earlobe color of Jiangshan black-bone chickens have been reported to be caused by the combined effects of melanin and collagen. In this study, we conducted LC-MS untargeted metabolomics and 16S rDNA diversity sequencing on the cecal contents of two types of earlobes: peacock green (Blue and Green group) and dark reddish-purple (Black group). The metabolomic sequencing identified a total of 747 differential metabolites (DMs), in which the metabolites were primarily enriched in tyrosine and tryptophan metabolism pathways between peacock green and dark reddish-purple earlobes. There were 15 different bacterial taxa among the three groups of earlobes at the genus level, and correlation analysis between metabolites and microbes revealed that the DMs between peacock green and dark reddish-purple earlobes were positively correlated with the different bacterial taxa. In short, there are differences in gut microbiota and metabolites between Jiangshan black-bone chickens with peacock green earlobes and those with dark reddish-purple earlobes. Our results suggest that the bacterial phyla Firmicutes and Bacteroidota may influence melanin synthesis by affecting tryptophan metabolism, induced by 5-Methoxyindoleacetate, and tyrosine metabolism, induced by maleylacetoacetic acid and maleic acid, leading to differences in earlobe color.

## Simple Summary

The differences in the earlobe color of Jiangshan black-bone chickens have been reported to be caused by the combined effects of melanin and collagen. In this study, we found differences in gut microbiota and metabolites between Jiangshan black-bone chickens with peacock green and dark reddish-purple earlobes. The gut microbiota influences melanin synthesis by affecting tryptophan and tyrosine metabolism, resulting in variations in earlobe color. Furthermore, metabolomic sequencing and microbial diversity analysis revealed a range of metabolites and microbial communities, providing a theoretical basis for explaining the earlobe color differences in Jiangshan black-bone chickens.

## 1. Introduction

The earlobes of chickens are elliptical or semi-circular in shape, with many lines and irregularities, and hang below the ear hole of chickens, playing a visual signal role in age, mental vitality, and reproduction. Earlobe color is a quantitative trait in chickens and serves as a characteristic of the breed [1,2,3]. Jiangshan black-bone chickens, known for their medicinal value, are famous for their unique peacock green earlobes, which exhibit a bright iridescent blue or green hue [4,5]. There are two types of earlobes in Jiangshan black-bone chickens: peacock green and dark reddish-purple. The peacock green color is divided into two groups: dark peacock green (Blue group) and light peacock green (Green group). Additionally, some Jiangshan black-bone chickens have an earlobe color that matches the dark reddish-purple of their skin, with a slightly darker tint (Black group) [6]. In breeding practices, peacock green earlobes are often a key selection criterion.

Over the past few decades, research on chicken earlobe color has been relatively limited, especially regarding the distinct earlobe color of Jiangshan black-bone chickens. Our previous studies focused on the earlobe tissue of chickens, revealing that the earlobe color differences in Jiangshan black-bone chickens are due to variations in melanin and collagen from the perspectives of transcriptome, proteome, metabolome, and pigment deposition [6,7]. However, no research has yet explored the differences in gut metabolism and microbial communities among chickens with different earlobe colors. The gut microbiota can convert host nutrients into complex metabolites, and these metabolites play a crucial role in the variation of biological traits [8]. Mechanisms such as providing metabolic energy, promoting biosynthesis, and modifying signal proteins [9] can influence pigment formation and protein synthesis, thereby leading to the development of different earlobe color traits.

Therefore, this study conducted LC-MS-based untargeted metabolomics and 16S rDNA diversity sequencing on the cecal feces of Jiangshan black-bone chickens with three different earlobe colors. The aim was to identify differences in gut metabolites and to search for the biomarkers responsible for earlobe color variation, thereby gaining a deeper understanding of the mechanisms behind earlobe color formation in Jiangshan black-bone chickens.

## 2. Materials and Methods

### 2.1. Animal Material and Fecal Collection

Four 360-day-old Jiangshan black-bone chickens with blue, green, and black earlobes were selected and were fed with a basal diet and raised in cages with free access to feed and water at Jiangshan Lanfeng Poultry Co., Ltd (Zhejiang, China). The cecal contents were collected and stored at −80 °C. The chicken slaughter plan was processed and approved in accordance with the requirements of the Animal Protection and Utilization Committee of Zhejiang Agriculture and Forestry University, with approval number ZAFUAC202401.

### 2.2. LC-MS Metabolome

Fecal samples were subjected to LC-MS/MS analysis using a Thermo Fisher Scientific UHPLC-Q Exactive HF-X system (Thermo Fisher, Waltham, MA, USA). This analysis was performed by Majorbio Bio-Pharm Technology Co., Ltd., (Shanghai, China).

The raw LC-MS data were matched against the public metabolite databases HMDB (http://www.hmdb.ca/, accessed on 28 March 2024) and Metlin (https://metlin.scripps.edu/, accessed on 28 March 2024) to obtain metabolite information. Partial Least Squares Discriminant Analysis (PLS-DA) was performed on the preprocessed data matrix using the ropls package (Version 1.6.2) in R. Significantly different metabolites were identified based on the variable importance in projection (VIP) scores obtained from the OPLS-DA model and Student’s *t*-test *p*-values. Metabolites with VIP > 1 and *p* < 0.05 were considered significantly different. These differential metabolites were then annotated with metabolic pathways using the KEGG database (https://www.kegg.jp/kegg/pathway.html, accessed on 12 April 2024).

### 2.3. 16S rDNA Sequencing

The total microbial genomic DNA from the fecal microbiota was extracted using an E.Z.N.A.^®^. Soil DNA kit (Omega Bio-tek, Norcross, GA, USA) according to the manufacturer’s instructions. The DNA concentration and purity were determined using NanoDrop 2000 (Thermo Fisher, Waltham, MA, USA). The hypervariable region V3-V4 of the bacterial 16S rRNA gene was amplified with primer pairs 338F (5′-ACTCCTACGGGAGGCAGCAG-3′) and 806R (5′-GGACTACHVGGGTWTCTAAT-3′) [10] by T100 Thermal Cycler PCR thermocycler (BIO-Rad, Hercules, CA, USA). Purified amplicons were pooled in equimolar amounts and paired-end sequenced on an Illumina Nextseq 2000 platform (Illumina, San Jose, CA, USA). The raw data analysis was performed by Majorbio Bio-Pharm Technology Co. Ltd. (Shanghai, China). Fastp [11] (https://github.com/OpenGene/fastp, version 0.19.6, accessed on 15 May 2024) was used for quality control, FLASH [12] (http://www.cbcb.umd.edu/software/flash, version 1.2.11, accessed on 15 May 2024) was used for splicing, and DADA2 [13] plugin was used for noise reduction processing.

Qiime2 [14] (https://qiime2.org/, version 3.5.3, accessed on 12 June 2024) was used for species taxonomic analysis on microorganisms and the calculation of beta diversity distance matrices. Principal coordinate analysis (PCoA) based on the Bray–Curtis distance algorithm was used to test the similarity of microbial community structure between samples. Python 2.7 was used to analyze community composition. The Wilcoxon rank-sum test (*p* < 0.05) was performed to determine the bacterial groups with significant differences in abundance. The metagenomic function was predicted by PICRUSt2 [15] (version 2.2.0).

### 2.4. Correlation Analysis between Metabolites and Microbial Expression

The Spearman rank correlation coefficients between the top 50 species at the taxonomic level and the top 50 metabolites in abundance were calculated using the scipy.stats package in Python (version 2.7.10).

### 2.5. Statistical Analysis

We considered *p* < 0.05 to be statistically significant. * *p* < 0.05 was considered significant in difference; ** *p* < 0.01 and *** *p* < 0.001 were considered extremely significant in difference.

## 3. Results

### 3.1. Differences in Fecal Metabolites Among Chickens with Different Earlobe Colors

**Figure 1:**
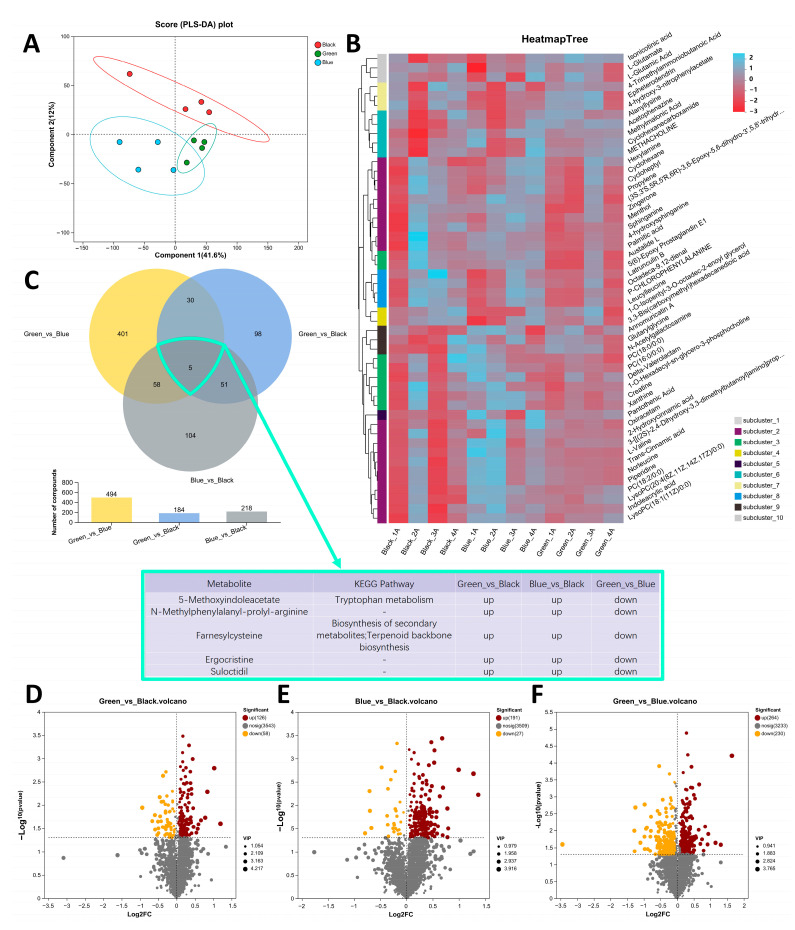
Differential metabolites (DMs) in feces of three types of earlobes. (**A**) PLS-DA analysis of three types of earlobes. (**B**) Cluster heatmap of fecal metabolites from three types of earlobes. (**C**) Venn diagram of DMs in three types of earlobes. (**D**–**F**) Volcano plots of DMs in Green_vs._Black (**D**), Blue_vs._Black (**E**), and Green_vs._Blue groups (**F**). (Black_1A, Black_2A, Black_3A, and Black_4A belong to the Black group; Blue_1A, Blue_2A, Blue_3A, and Blue_4A belong to the Blue group; and Green_1A, Green_2A, Green_3A, and Green_4A belong to the Green group. The screening threshold of DMs was *p* < 0.05 and VIP > 1).

To investigate the differences in metabolites in feces, we performed metabolomics on the feces of chickens with different earlobe colors. All raw data were deposited in the CNCB OMIX database (accession number OMIX007272). The number of raw metabolites identified in the positive ion mode was 2164, and in the negative ion mode was 2008. After data preprocessing, there were 1965 metabolites in the positive ion mode and 1759 in the negative ion mode (Supplementary Table S1). PCA analysis revealed significant overall metabolic differences among the three earlobe colors and low variability among samples within each group (Figure 1A), proving that the model could be used for subsequent research. The metabolic expression patterns of the three earlobe colors are shown in Figure 1B. A total of 747 differential metabolites (DMs) were detected (Figure 1C), with 184 between the Green and Black groups (Figure 1D), 218 between the Blue and Black groups (Figure 1E), and 494 between the Green and Blue groups (Figure 1F). Among them, there were five common DMs across the three groups, namely 5-Methoxyindoleacetate, N-Methylphenylalanyl-prolyl-arginine, farnesylcysteine, ergocristine and suloctidil (Figure 1C).

**Figure 2:**
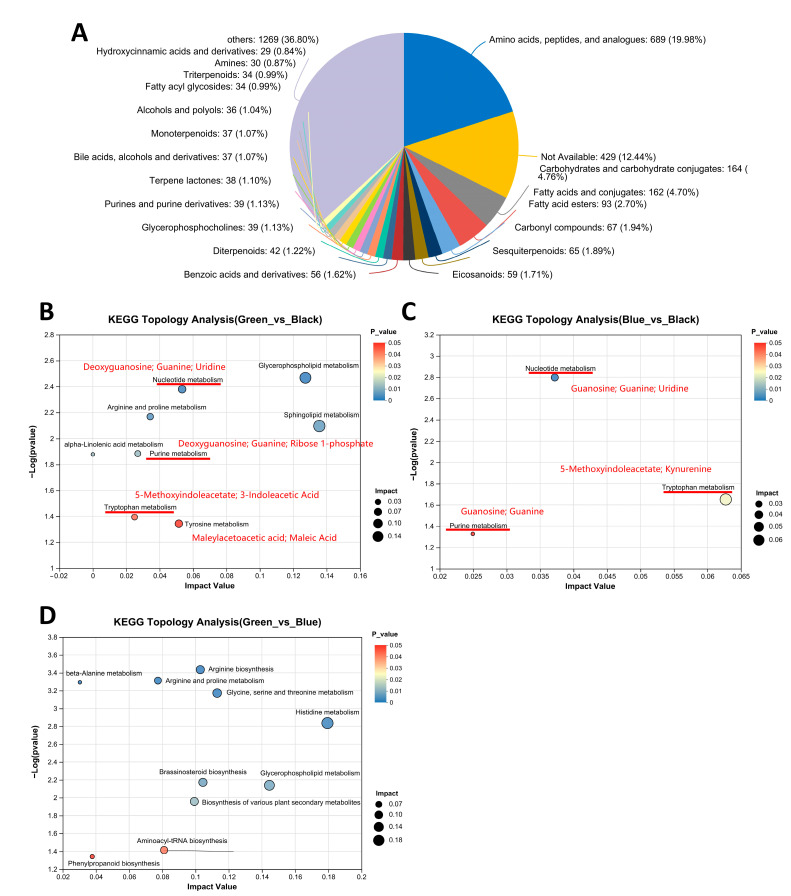
The functions of fecal metabolites in three types of earlobes. (**A**) HMDB compound classification of three types of earlobes. (**B**–**D**) KEGG topology analysis of DMs in Green_vs._Black (**B**), Blue_vs._Black (**C**), and Green_vs._Blue groups (**D**). The red lines in (**B**,**C**) showed the 3 common pathways of Green_vs_Black and Blue_vs_Black.

The HMDB compound classification of metabolites in earlobes revealed a higher proportion of amino acids, peptides, and analogs (Figure 2A). KEGG topology analysis was conducted on the DMs of three earlobes, and it was found that there were eight significantly different KEGG pathways in the Green_vs._Black groups (Figure 2B, Supplementary Table S2), three in Blue_vs._Black groups (Figure 2C, Supplementary Table S2), and 10 in Green_vs._Blue groups (Figure 2D, Supplementary Table S2). There were three common pathways in Green_vs._Black and Blue_vs._Black groups, namely nucleotide metabolism, purine metabolism, and tryptophan metabolism (Figure 2B,C). The DMs enriched in these pathways were deoxyguanosine, guanosine, guanine, uridine, ribose 1-phosphate, 5-Methoxyindoleacetate, 3-Indoleacetic acid, and kynurenine (Figure 2B,C). Additionally, tyrosine metabolism was enriched in the Green_vs._Black groups, and the DMs were maleylacetoacetic acid and maleic acid (Figure 2B).

### 3.2. Differences in Gut Microbiota Among Chickens with Different Earlobe Colors

To investigate the differences in gut microbiota, we performed 16S rDNA sequencing on the cecal feces of chickens with different earlobe colors. All raw data were deposited in the CNCB GSA database (accession number CRA013533). A total of about 916 thousand raw reads (275 million raw bases) were obtained through sequencing, with an average sequence length of 418 bp for each sample (Supplementary Table S3). After denoising with DADA2, approximately 434 thousand sequences were generated, with 27,568–49,061 sequences per sample, resulting in 8,656 Amplicon Sequence Variants (ASVs) (Supplementary Table S3).

**Figure 3:**
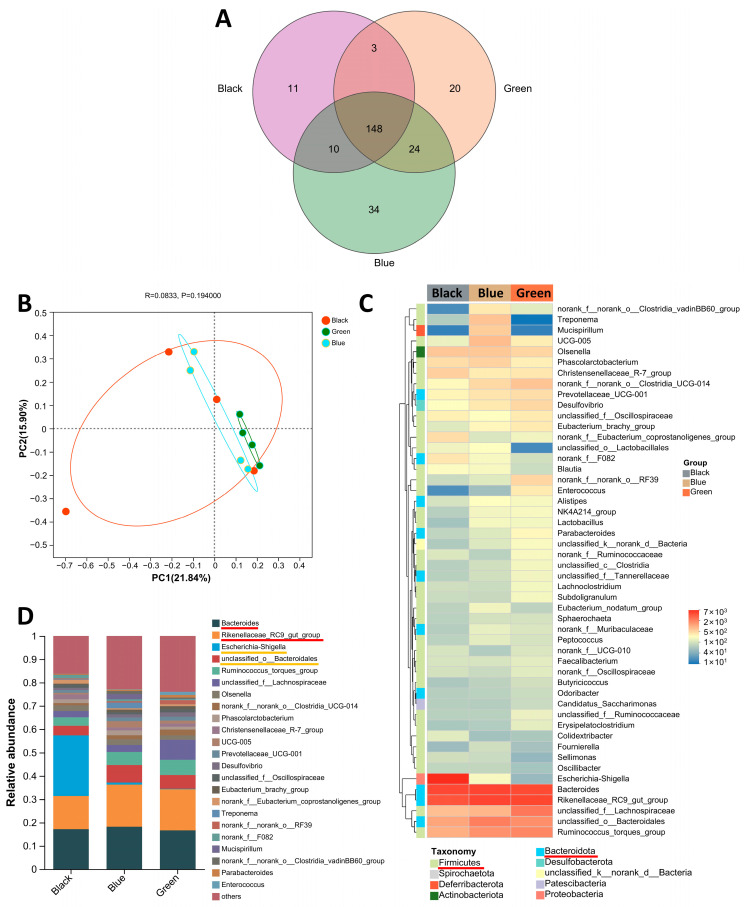
Differential microorganisms in feces of three types of earlobes. (**A**) Venn diagram of microorganisms in three types of earlobes. (**B**) PCoA analysis of microorganisms in three types of earlobes. (**C**) Community heatmap of fecal microorganisms from three types of earlobes. The red lines in (C) indicate the two species with the highest abundance of earlobe color, most of which belong to Firmicutes and Bacteroidota. (**D**) Community bar chart of fecal microorganisms from three types of earlobes. The red lines in (**D**) indicate Bacteroides and Rikenellaceae_RC9_gut_group were common dominant gates except for Black group; The orange lines in (**D**) indicate Escherichia-Shigella and Bacteroidales were strains with large differences in abundance. (Black_1A, Black_2A, Black_3A, and Black_4A belong to the Black group; Blue_1A, Blue_2A, Blue_3A, and Blue_4A belong to the Blue group; and Green_1A, Green_2A, Green_3A, and Green_4A belong to the Green group).

PCoA analysis showed differences in bacterial community structure among the three groups (Figure 3B). At the genus level, there were 148 common species in the three groups, with 11 unique species in the Black group, 34 unique species in the Blue group, and 20 unique species in the Green group (Figure 3A). The community heatmap displays the top 50 species with the highest abundance of the three earlobe colors at the genus level, with the majority of strains belonging to Firmicutes and Bacteroidota (Figure 3C). The community bar chart displays the bacterial composition of the three earlobe colors at the genus level (Figure 3D). Among the three earlobe colors, Bacteroides and the Rikenellaceae_RC9_gut_group were common dominant gates, except for the Black group (Figure 3D, red bar). The relative abundance of Escherichia–Shigella was higher in the Black group (25.88%) and lower in the Green group (0.18%) and Blue group (0.86%); the relative abundance of Bacteroidales was higher in the Blue group (7.53%) and Green group (5.84%) and lower in the Black group (4.15%) (Figure 3D, orange bar).

**Figure 4:**
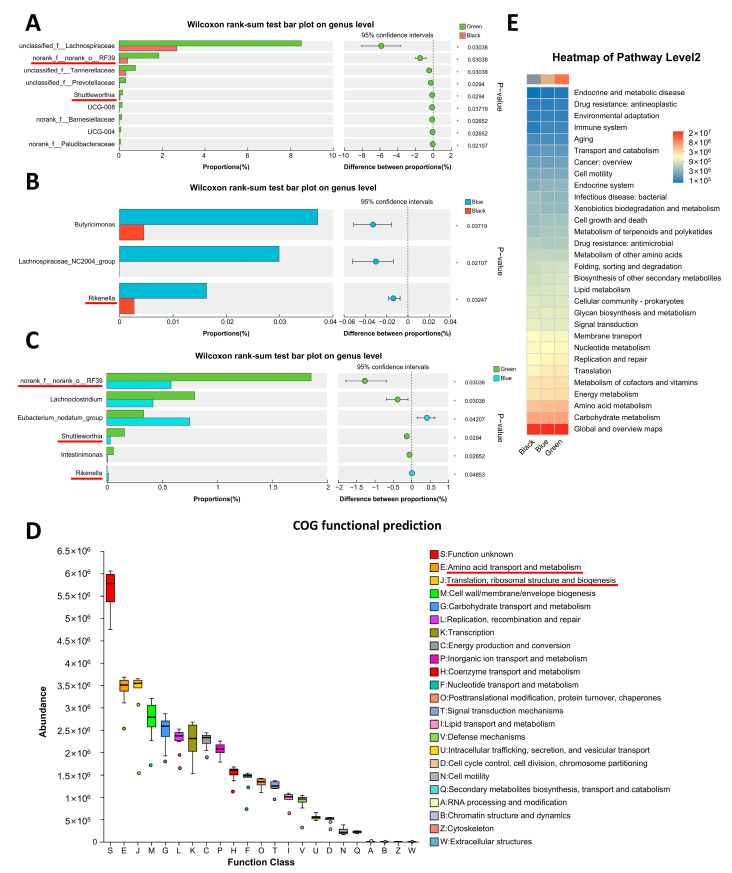
Differential microorganisms and their functions in three types of earlobes. (**A**–**C**) Differential microorganisms in Green_vs._Black (**A**), Blue_vs._Black (**B**), and Green_vs._Blue groups (**C**). The red lines indicate common microorganisms of three types of earlobes. (**D**) COG function prediction of microorganisms from three types of earlobes. The red lines indicate the main microbial functions of the three earlobe colors. (**E**) KEGG heatmap of microorganisms from three types of earlobes.

There were significant differences in genus-level microorganisms between earlobe colors (two-sided unpaired Wilcoxon test, *p* < 0.05). There were nine microorganisms with significant abundance differences (*p* < 0.05) between the Green and Black groups (Figure 4A), three microorganisms (*p* < 0.05) between the Blue and Black groups (Figure 4B), and six microorganisms (*p* < 0.05) between the Green and Blue groups (Figure 4C). They shared common microorganisms such as RF39, Shuttleworthia, and Rikenella, which may be biomarkers of earlobe color (Figure 4A–C). By predicting the COG function of all microorganisms in the three groups through PICRUSt2, it was found that the microbial functions of the three earlobe colors are mainly amino acid transport and metabolism, as well as translation, ribosomal structure, and biogenesis (Figure 4D). The KEGG functional heatmap shows that there were functional differences such as cell growth and death, nucleotide metabolism, and amino acid metabolism between earlobes (Figure 4E).

### 3.3. Correlation Analysis Between the Cecal Fecal Metabolites and Gut Microbiota in Chickens with Different Earlobe Colors

**Figure 5:**
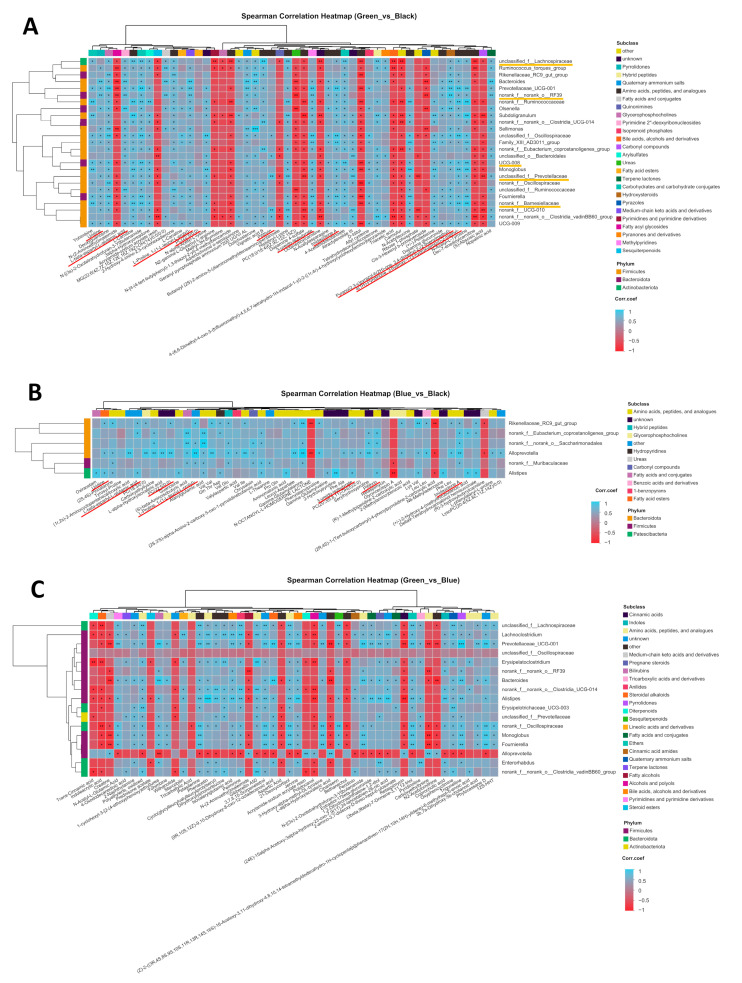
Correlation heatmap between the cecal fecal metabolites and gut microbiota. (**A**–**C**) Correlation heatmap for Green_vs._Black (**A**), Blue_vs._Black (**B**), and Green_vs._Blue groups (**C**). The red lines represent DMs between different earlobes, and the orange lines represent differential microbial communities between different earlobes. We considered *p* < 0.05 to have significant correlation between the microbial communities and the fecal metabolites. * *p* < 0.05, ** *p* < 0.01, and *** *p* < 0.001.

Microorganisms can produce changes in metabolites by synthesizing, regulating, or degrading endogenous substances, which, in turn, can have a certain impact on the functions of the organism. We conducted a correlation analysis between the cecal fecal metabolites and microorganisms in chickens with three different earlobe colors and used Spearman correlation analysis to study the relationship between the DMs and differential bacteria (genus level). We found a significant correlation between microbial communities and fecal metabolites (Figure 5A–C, Supplementary Table S4). Given the earlobe color classification of Jiangshan black-bone chickens, we focused on the correlation between 56 common DMs (highlighted in red in Figure 5A,B) and 12 differential microbial communities (highlighted in orange in Figure 5A) of peacock green earlobes vs. dark reddish-purple earlobes (Green_vs._Black, Blue_vs._Black groups). The results showed a positive correlation between these DMs and microorganisms (Figure 5A,B). Specifically, there was a positive correlation between metabolites such as PC(22:5/0:0), 3-ureido-isobutyrate, imidazolone A, L-Proline, 1-(1-L-leucyl-L-prolyl)-, 4(1H)-Pyridinone, 3-hydroxy-1-(3-hydroxypropyl)-2-methyl-, L-beta-aspartyl-L-glutamic acid, pyrano(2,3-c)pyrazol-6(2H)-one, 3,4-dimethyl-2-(2-thienylmethyl)-, pifithrin, and uridine, and microorganisms such as Lachnospiraceae, RF39, UCG-008, Prevotellaceae, and Barnesiellaceae (Figure 5A,B). This indicates that these differential microbial communities may be biomarkers responsible for the color differences between peacock green earlobes and dark reddish-purple earlobes and that they produce functional differences by affecting these DMs. Additionally, the HMDB compounds of these DMs were mostly classified as amino acids, peptides, and analogs, while most of the differential communities belonged to Firmicutes and Bacteroidota (Figure 5A).

## 4. Discussion

In previous research, we discovered that there is a tyrosine-induced melanin difference between the peacock green earlobe tissue and the dark reddish-purple earlobe tissue in Jiangshan black-bone chickens [6,7]. However, it remains unclear whether and how this metabolic pathway is regulated by microorganisms.

Through fecal metabolomics analysis, we identified a common DM, 5-Methoxyindoleacetate, among the three groups, which originates from the tryptophan metabolic pathway (Figure 1C). When comparing the DMs between the peacock green earlobes (Blue and Green groups) and the dark reddish-purple earlobes (Black group), we were once again surprised to find a shared KEGG pathway between the earlobes—tryptophan metabolism (Figure 2B,C). This indicates that there is a tryptophan metabolism difference between the peacock green and dark reddish-purple earlobes induced by 5-Methoxyindoleacetate. Additionally, the tyrosine metabolism pathway was enriched between the Green and Black groups, with DMs identified as maleylacetoacetic acid and maleic acid (Figure 2B). This further confirms the presence of tyrosine metabolism differences between the peacock green and dark reddish-purple earlobes. Tyrosine is a key precursor for melanin synthesis as it is converted into dopa and dopaquinone through a series of enzymatic reactions, particularly catalyzed by tyrosinase, eventually leading to melanin formation [16]. Although tryptophan does not directly participate in melanin synthesis, it can indirectly influence melanin synthesis and metabolism by being converted into 5-hydroxytryptamine (a precursor to melatonin) [17,18,19,20] and nicotinamide (a form of vitamin B3) [21,22,23,24,25,26]. 5-hydroxytryptamine can, under certain conditions, be converted into tyrosine, which is a direct precursor for melanin synthesis [27,28]. Ahmad et al. [29] found that strain JA2 produced a brown pigment under the action of L-tryptophan. Therefore, the metabolites of tryptophan and tyrosine have a certain impact on melanin synthesis in Jiangshan black-bone chickens with different earlobe colors. Sanchez-Amat et al. [30] found that maleylacetoacetic acid can affect melanin formation by affecting tyrosine catabolism. Shen et al. [31] found that malic acid was involved in the tyrosine metabolism pathway. The DMs in both the peacock green earlobes (Blue and Green groups) and the dark reddish-purple earlobes (Black group) were also commonly enriched in the nucleotide metabolism and purine metabolism pathways (Figure 2B,C). Some intermediates produced during nucleotide metabolism, such as guanosine triphosphate (GTP), can serve as substrates or regulators for tyrosinase [32,33,34]. Additionally, purine metabolism products like uric acid can influence tyrosinase activity, affecting the conversion of tyrosine to melanin and thereby impacting melanin synthesis and pigment deposition [35,36].

Through fecal microbiome diversity analysis, we identified 12 different bacterial taxa between the peacock green earlobes (Blue and Green groups) and dark reddish-purple earlobes (Black group) (Figure 4A,B), along with differences in microbiome functions related to amino acid and nucleotide metabolism (Figure 4E). This aligns with the metabolomic results, suggesting that the gut microbiota may influence earlobe color formation by affecting the synthesis of metabolites.

By correlating fecal metabolites with gut microbiota, we identified a positive correlation between nine DMs and five differential bacterial taxa between the peacock green earlobes (Blue and Green groups) and dark reddish-purple earlobes (Black group) (Figure 5A,B). This suggests that these bacterial taxa are biomarkers influencing earlobe color differences by regulating metabolite synthesis. Most of these DMs are related to amino acids, and the differential bacterial taxa primarily belong to Firmicutes and Bacteroidota (Figure 5A,B). Firmicutes are a phylum composed of various Gram-positive bacteria, one of the two most abundant groups in the gut microbiome, playing a significant role in digestion, the immune system, and metabolic processes [37]. Bacteroidota is a phylum of Gram-negative bacteria widely present in animal guts, mainly involved in breaking down complex carbohydrates and maintaining gut microbiota balance [38]. Some studies have suggested that an imbalance in the gut microbiota is associated with skin inflammation and abnormal pigmentation [39,40]. Tsai et al. [41] found that Lactobacillus plantarum (Firmicutes) reduced skin melanin synthesis by improving skin microbiota. Ma et al. [42] found that *Bacteroidetes* can affect skin melanin synthesis, while Lee et al. [43] discovered that Flavobacterium (*Bacteroidetes*) has the potential for melanin production.

## 5. Conclusions

This study found that there are differences in gut microbiota and metabolites between Jiangshan black-bone chickens with peacock green earlobes and those with dark reddish-purple earlobes. The bacterial phyla Firmicutes and Bacteroidota may influence melanin synthesis by affecting tryptophan metabolism, induced by 5-Methoxyindoleacetate, and tyrosine metabolism, induced by maleylacetoacetic acid and maleic acid, leading to differences in earlobe color.

## Data Availability

The original data in the article can be obtained directly from the corresponding author. All the 16S rDNA sequence data have been deposited in CNCB (China National Center for Bioinformation) Genome Sequence Archive (GSA, https://ngdc.cncb.ac.cn/gsub/, accessed on 11 May 2024) and are accessible through GSA series accession number CRA013533: https://ngdc.cncb.ac.cn/gsa/, accessed on 11 May 2024. All the metabolome sequence data have been deposited in CNCB (China National Center for Bioinformation) Open Archive for Miscellaneous Data (OMIX, https://ngdc.cncb.ac.cn/omix/, accessed on 4 September 2024) and are accessible through OMIX series accession number OMIX007272.

## Supplementary Materials

The following supporting information can be downloaded at https://www.mdpi.com/article/10.3390/ani14213060/s1. Supplementary Table S1: Information on LC-MS metabolome in each sample. Supplementary Table S2: KEGG function of DMs between three groups. Supplementary Table S3: Information on 16S rDNA sequencing in each sample. Supplementary Table S4: The correlation between metabolites and microbial communities.
